# Positive Psychology Insights on the Effects of Spirituality on Shared Decision-Making in Patients with Chronic Heart Failure: The Chain-Mediated Effects of Benefit-Finding and Decision Self-Efficacy

**DOI:** 10.3390/healthcare13101188

**Published:** 2025-05-19

**Authors:** Zitian Liu, Weiyue Zhou, Yinglan Wu, Yuxin Zhou, Weimin Jiang

**Affiliations:** 1 The First Clinical Medical School, Nanjing University of Chinese Medicine, No. 138 Xianlin Avenue, Nanjing 210023, China; 039316112@njucm.edu.cn (Z.L.); zwy007@njucm.edu.cn (W.Z.); evelyn816@njucm.edu.cn (Y.W.); zyx0701@njcum.edu.cn (Y.Z.); 2 Department of Cardiology, Affiliated Hospital of Nanjing University of Chinese Medicine, No. 155 Hanzhong Road, Nanjing 210023, China

**Keywords:** heart failure patients, shared decision-making, spirituality, benefit-finding, decision self-efficacy, chain mediation

## Abstract

**Background**: As the terminal stage of cardiovascular disease, heart failure (HF) has garnered significant attention due to its recurrent nature, high mortality rates, and substantial medical burden. Shared decision-making (SDM) is an innovative strategy to improve medication adherence. From positive psychology insights, the effects on spirituality, benefit-finding (BF), decision self-efficacy, and patient engagement in SDM remain unexplored. **Methods**: This quantitative cross-sectional study was conducted from January 2023 to September 2024 at a hospital in Jiangsu. Data on general information, spirituality, BF, decision self-efficacy, and SDM were collected from 387 patients with chronic heart failure. **Results**: Spirituality was significantly associated with SDM (β = 0.8839, *p* < 0.001). BF played a mediating role in the relationship between spirituality and SDM (β = 0.2020, 95% CI: 0.0058–0.0261), accounting for 22.9% of the total effect. Decision self-efficacy was identified as a mediator in this relationship (β = 0.2636, 95% CI: 0.0120–0.0284), accounting for 29.8%. In addition, both BF and decision self-efficacy exhibited a chain mediation effect on the association between spirituality and SDM (β = 0.1451, 95% CI: 0.0061–0.0162), and the total indirect effect accounted for 69.1%. **Conclusions**: This study is the first to demonstrate that spirituality has significant direct and indirect effects on SDM, and it also reveals the underlying psychological mechanisms. Spiritual support services, BF intervention, and enhancing patients’ decision self-efficacy can promote their participation in SDM. These findings highlight the role of positive psychology in promoting SDM, showing potential contribution to promoting medication adherence in HF patients.

## 1. Introduction

Heart failure (HF) represents a severe manifestation or terminal stage of various cardiovascular diseases. As the global population ages and sedentary lifestyles become more prevalent, the incidence of cardiovascular diseases is projected to increase by 90% in the next 25 years [1]. Patients with HF still face high medical costs due to recurrent attacks. The average annual medical cost of patients with HF ranged from USD 2128 to USD 30,638 approximately in eleven countries [2]. Additionally, the mortality risk remains significant. Data from China in 2024 indicates that the three-year post-discharge mortality rate for HF patients is 28.2% [3]. According to 27 studies, Lan et al. found that the one-year post-discharge mortality rate for HF patients had reached 29% [4].

Guideline-recommended anti-heart failure drugs can substantially decrease HF-related mortality; thus, medication compliance is crucial for controlling recurrent HF [5]. To maximize patient benefits from anti-HF drugs, it is critical to enhance medication adherence and achieve the target dose as recommended by clinical guidelines [5]. At present, shared decision-making (SDM) has been proven to be a new effective strategy to improve medication adherence [6]. SDM represents a people-centered healthcare service wherein healthcare professionals (HCPs) and patients share information, discuss options, and choose treatment options based on the patient’s personal preferences [7]. Decision self-efficacy refers to a patient’s confidence in making informed choices during healthcare. It encourages patients to actively engage in treatment and nursing decision-making [8]. For SDM to occur effectively, patients must possess both the intention and the confidence to actively engage in the decision-making process. A study of the readiness of patients to carry out SDM identified eight factors that enable them to carry out SDM. In addition to self-efficacy and emotional distress, SDM factors include the ability to imagine how treatment options affect personal life. Such imagination is the key thinking skill of patients in SDM [9]. Systematic reviews of patient characteristics related to SDM have consistently shown that patients with positive attitudes and high self-efficacy are more likely to engage in SDM [10]. The role of variables from positive psychology in SDM cannot be ignored.

At present, the relationship between positive psychology and SDM has gradually attracted attention. Researchers have explored the relationship among positive psychology, SDM, employment, and disability through literature reviews and found that the aim of both positive psychology and SDM is to enhance well-being and human flourishing [11]. At the same time, it was proposed that when planning SDM in the context of career development activities, it is necessary to adopt interventions derived from positive psychology and PERMA models to support positive emotions and participation in SDM [11]. PERMA includes elements such as meaning, positive connection, and so on [12]. Meaning also exists in the concepts of positive psychology, such as benefit-finding (BF) [13], hope [14], and spirituality [15]. Positive connection is a key component of spirituality [16]. Therefore, we hypothesize that BF and spirituality are capable of facilitating SDM. However, the relationships and pathways among spirituality, BF, decision self-efficacy, and SDM have not been elucidated.

Spirituality is a core state that integrates one’s physical, mental, social, and intellectual dimensions, as well as overall health, while fostering connections with oneself, others, and the external environment [17]. Studies in the field of health have demonstrated that spirituality is positively associated with self-efficacy and can serve as a predictor of individual self-efficacy [18]. Decision efficacy is the specific form of individual self-efficacy in decision-making contexts [19]. A correlation between religiously integrated spirituality and decision efficacy has been demonstrated in a study on ICU patients [20]. Research has also demonstrated the role of spirituality in enhancing medication adherence, promoting smoking cessation, and facilitating lifestyle modifications [21,22]. According to the Britannica Dictionary, spirituality is characterized by an individual’s inner spiritual qualities and experiences [23]. Swihart et al. [24] found that patients pursue spiritual beliefs while making medical decisions. The evidence above indicates that spirituality may have a positive relationship with medical decision-making. Ozdemir et al. also demonstrated that any form of decision-making participation in patients with HF was related to the spiritual level [25].

Mahoney suggested that the benefits of spirituality are inspiring life purpose and meaning [26]. Meaning in life involves in the concept of BF [13]. In the field of health, BF refers to the process by which individuals actively seek positive meaning in life and growth opportunities in disease events or states by adjusting cognition and psychological adaptation [27,28]. At present, several theoretical models of BF have showed that finding meaning in life contributes to individual BF [29,30]. The role of spirituality on BF has been well proven. Studies have shown that BF and rumination are the chain mediators between spirituality and medication adherence [31]. The direct effect of BF on individual self-management has been demonstrated in both HF and COPD patients [32,33]. Decision-making ability is the core ability of self-management [34], and self-efficacy is an important component of self-management measurement scales [35]. In addition, qualitative research shows that BF has provided individuals with motivation and hope [36]. In SDM, active patients are more able to participate in SDM and improve the quality of their decision-making. Hope is the motivation of the individual to produce behavior change [37]. Therefore, it is reasonable to hypothesize that BF may influence decision efficacy and SDM, potentially acting as a direct mediator between spirituality and SDM. Although it has been proven that BF changes health behaviors through self-efficacy in medication compliance and healthy eating behaviors [38], so far no studies have mentioned BF in SDM concerning chronic disease management.

This study explores the chain-mediating effect of BF and decision self-efficacy between spirituality and SDM. This study offers evidence for applying spiritual support services and BF in SDM. Hoping for high-quality SDM helps patients with HF realize medication compliance. The promotion effect of shared decision-making on patients’ health behavior has been confirmed, so the promotion of shared decision-making makes a potential contribution to improving medication adherence in patients with HF.

From the above, the following assumptions can be made:

**H1.**  *Spirituality has a positive association with SDM in HF patients.*

**H2a.**  *BF acts as a mediating factor between SP and SDM.*

**H2b.**  *Decision self-efficacy acts as a mediating factor in the positive associations between SP and SDM.*

**H3.**  *BF and decision self-efficacy act as chain-mediating factors in the positive associations between SP and SDM.*

## 2. Materials and Methods

### 2.1. Participants and Procedure

A study of anti-heart failure drugs based on guidelines was conducted in Jiangsu Provincial Hospital of Chinese Medicine in China. The study received clinical trial registration (ID: ChiCTR2200060678). The ethical lot number is 2022NL-032-01. This research investigates the impact of positive psychology components on SDM.

During the period from January 2023 to September 2024, the convenience sampling method was used in this study. Patients diagnosed with chronic heart failure (CHF) were recruited through posters in the outpatient and inpatient departments of Jiangsu Provincial Hospital of Chinese Medicine. The diagnostic criteria for CHF refer to the 2021 ESC Guidelines for the diagnosis and treatment of acute and chronic heart failure [39]. The diagnosis was made by cardiologists. Inclusion criteria: (1) age ≥ 18 years old; (2) New York Heart Association Classification of Heart Failure (NYHA classification) class II–IV, resting heart rate > 55 beats per minute, blood pressure > 90/60 mmHg; (3) using oral anti-heart failure medications; (4) stable condition, capability of going to the hospital independently, regularly following up, self-monitoring heart rate and blood pressure at home; (5) patients without dementia (dementia evaluated according to education level and Mini-Mental State Examination scores [40]) and with the ability to complete the questionnaire independently or with assistance. Exclusion criteria: (1) patients with severe psychiatric symptoms; (2) patients with severe hepatic and renal insufficiency, malignant tumors, or other systemic serious primary diseases to avoid confounding factors and ethical risks.

According to f^2^ = 0.10, α = 0.05, β = 0.95, and 19 independent variables, the minimum sample size calculated by G. Power is 318. To ensure a valid sample size, we aimed to recruit at least 400 subjects. A total of 456 people were interested in the project, and eventually 400 people participated in the study. All participants volunteered to participate in the trial by signing an informed consent form. The questionnaire was designed to take 20–30 min. The study involved a total of five questionnaires to capture patients’ general information, the level of spirituality, BF, decision self-efficacy, and the level of SDM involvement. A total of 400 participants completed the above baseline information, with 387 valid questionnaires. The effective recovery rate was 96.75%.

### 2.2. Data Collection and Tools

#### 2.2.1. Sociodemographic and Health Data

Demographic information and clinical characteristics were collected using a self-made general information form.

#### 2.2.2. The Functional Assessment of Chronic Illness Therapy-Spiritual Scale (FACIT-Sp-12)

FACIT-Sp-12 was translated into Chinese in 2016 (Cronbach’s α = 0.831) [41]. The scale includes total of 12 items. The scoring of items follows a 5-point Likert scale, where “not at all consistent” receives a score of 0 and “very consistent” receives a score of 4. The fourth and fifth items are scored in the reverse direction, while the other 10 items are scored in the forward direction. The scale is self-rated by patients, and the total score ranges from 0 to 48. The level of spirituality is divided into three levels: low (<24 points), medium (24–35 points), and high (≥36 points). The higher the score, the higher the level.

#### 2.2.3. The Revised Chinese Version of Benefit-Finding Scale (BFS-C)

The study used the BFS-C revised by Bian Jing (Cronbach’s α = 0.933) [42]. Patients are assessed in five dimensions (including acceptance, family relationships, personal growth, social relationships, and health behaviors) with 22 items. Scoring is on a 5-point Likert scale, where a score of 1 means “not at all” and a score of 5 means “very much”. The scale has a total score of 22–110, with higher scores indicating more perceived benefit to the patient.

#### 2.2.4. Decision Self-Efficacy Scale (DSES)

The DSES was written by O’Connor et al. [43] and translated into Chinese by Wang Sitong et al. (Cronbach’s α = 0.918) [44]. The scale is used to assess how confident patients are in making informed choices while receiving healthcare and has been used in the studies of SDM [45]. The 11-item scale is based on a 5-point Likert scale ranging from “very low self-confidence” to “very high self-confidence” on a scale of 0–4. The 11 item scores are added together to take the mean and multiplied by 25 to convert to a score of 0–100. The higher the score, the higher the level of the patient’s decision-making self-efficacy.

#### 2.2.5. 9-Item Shared Decision-Making Questionnaire (SDM-Q-9)

The SDM-Q-9 assesses the behavior of both doctors and patients during SDM from the patient’s perspective. This scale has been administrated in the context of cardiovascular disease (Cronbach’s α = 0.96) [46]. Luo Bihua et al. translated it into Chinese (the Cronbach’s α is 0.945 among Chinese patients) [47]. The scale includes 9 items. Each item is scored on a 6-point Likert scale (completely disagree = 0, basically disagree = 1, somewhat disagree = 2, somewhat agree = 3, basically agree = 4, completely agree = 5), with scores ranging from 0 to 45. Raw scores were multiplied by 20/9 to convert to a score of 0 to 100, and a higher score indicates a higher level of patient participation.

### 2.3. Statistical Analysis

Descriptive statistics for sociodemographic and clinical information, spirituality scores, BF scores, DSES scores, and SDM scores were conducted by SPSS 26.0. Before conducting the data analysis, the Kolmogorov–Smirnov test was applied to assess the normality of the numerical variables. Before the parametric test, Levene’s test was used to examine homoscedasticity. If the variances were homogeneous, *t*-test or one-way analysis of variance (ANOVA) was used. If the variances were not homogeneous, Welch’s *t*-test or Welch’s ANOVA was used. The above-mentioned test were performed to compare the spirituality, BF, DSES, and SDM scores based on demographic variations. The correlations between spirituality, BF, DSES, and SDM were examined by Pearson correlation coefficients (r), and the significance of the r-values was tested. Multicollinearity among the variables was assessed by calculating tolerance and variance inflation factors (VIFs). After excluding that there was no multicollinearity problem between variables, a four-step hierarchical regression was conducted, entering potential confounders (Block 1), spirituality (Block 2), BF (Block 3), and DSES (Block 4). Model 6 of the PROCESS plug-in was used to examine the chain mediation effects. Bootstrapping with 5000 resamples was performed to estimate the mediation effects, generating bias-corrected 95% confidence intervals (CIs). A mediation effect was considered significant if the 95% CI excluded zero.

## 3. Results

### 3.1. Participants’ Sociodemographic and Clinical Characteristics

In this study, a total of 387 patients were included, with 66.1% being male and 33.9% being female. The mean age of participants was 65.23 ± 12.75 years (range: 30–90 years). In terms of marital status, only 18.9% of patients were single. The distribution of residence showed that 50.6% of the patients lived in cities. Among the patients, 32.3% had only received junior high school education or less, 56.3% had received senior high school education, and 11.4% had received university education or above. The monthly income of the patients included CNY ≤ 5000 (47.8%) and CNY > 5000 (52.2%). Only 10.6% of patients reported a religious affiliation. The BMI distribution of patients was as follows: BMI ≤ 18.5 (5.4%), 18.5 < BMI ≤ 22.9 (31.8%), 22.9 < BMI ≤ 24.9 (24.5%), 24.9 < BMI ≤ 30 (33.9%), BMI > 30 (4.4%). The duration of disease was as follows: ≤12 months (51.2%), 13–24 months (16.0%), 25–36 months (14.0%), and ≥37 months (18.9%). In terms of lifestyle, 53.2% of the patients were smokers. Furthermore, the comorbidities were distributed as follows: hyperuricemia (7.0%), hypertension (50.9%), hyperlipidemia (34.1%), and hyperglycemia (24.3%). A total of 16.0% of patients had a heart rate of ≥100 beats/minute. According to the NYHA grading of heart function, the proportion of patients with class II, III, and IV was 25.6%, 49.1%, and 25.3%, respectively.

In addition, the level of spirituality in patients with CHF differed in terms of residence and monthly income. The level of BF in patients with CHF varied in terms of educational attainment. There were differences in DSES among different ages, residence, and whether they had hyperuricemia. Moreover, the degree of SDM with CHF varied in terms of the NYHA grading of heart function and disease duration. Normality was assessed by the Kolmogorov–Smirnov test. All variables showed *p*-values of between 0.052 and 0.200 (*p* > 0.05), indicating that the data met the assumption of normality. Refer to Table 1 for details.

### 3.2. Descriptive Characteristics of Target Variables and Relationships Among Them

The descriptive characteristics of the target variables are shown in Table 2. The mean score of FACIT-SP-12 was 26.43 ± 6.74, at a moderate level; the mean score of BFS-C was 58.59 ± 16.31; the mean score of DSES was 62.53 ± 14.26; and the mean score of SDM-Q-9 was 62.12 ± 13.39. The normality of numeric variables was tested by the Shapiro–Wilk test. Moreover, scale scores for all target variables were divided by the number of scale entries to obtain the standardized score of M ± SD, which facilitated comparisons across scales.

### 3.3. Correlation Between SDM and Other Variables

As shown in Table 3, the average total scores of spirituality, BF, DSES, and SDM were 26.43 ± 6.74, 58.59 ± 16.31, 62.53 ± 14.26, and 62.12 ± 13.39, respectively. Additionally, the results of the Pearson correlation analyses indicated a significant positive correlation between spirituality and BF (*r* = 0.570, *p* < 0.01), as well as a significant positive correlation between BF and SDM (*r* = 0.450 *p* < 0.01). In addition, there was a significant positive correlation between DSES and SDM (*r* = 0.546, *p* < 0.01).

### 3.4. The Direct Effect of Variables on Predicting SDM

As shown in Table 4, in the first step, statistically significant covariates from univariate analyses (age, education level, religions, disease duration, HUA, HR, and NYHA classification) were included in Block 1, with SDM score as the dependent factor, which showed that Block 1 significantly accounted for 2.5% of the variance in SDM scores (F = 2.409, ΔR^2^ = 0.043, *p* < 0.05). When spirituality was added to Block 2, the results showed that BF significantly explained 21.2% of the SDM variance (F = 13.994, ΔR^2^ = 0.186, *p* < 0.001). In addition, when BF was added to Block 3, the model significantly explained 26.6% of the SDM variance (F = 16.554, ΔR^2^ = 0.055, *p* < 0.001). In Block 4, the addition of DSES helped improve the model by significantly increasing the explanatory power by 10.1% (F = 23.367, *p* < 0.001). Overall, the final model explained 36.7% of the SDM variance and demonstrated that higher spirituality (β = 0.137, *p* < 0.05), higher BF (β = 0.169, *p* < 0.001), and improved DSES (β = 0.394, *p* < 0.001) significantly predicted better SDM. In addition, the results of the multicollinearity test showed that the tolerance of all variables were greater than 0.1, and the variance inflation factors (VIFs) were all less than 2, indicating that there was no multicollinearity issue among the variables.

### 3.5. The Chain-Mediating Effect Test

**Figure 1:**
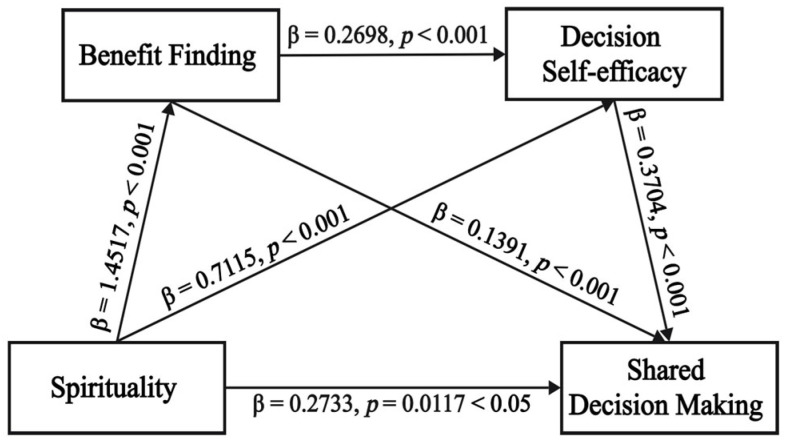
Chain mediation model of benefit-finding and decision self-efficacy between spirituality and shared decision-making of patients with chronic heart failure. Spirituality is the predictor, benefit finding and decision self-efficacy are the mediators, and shared decision-making is the outcome variable. (Note: *p* < 0.05 indicates statistical significance; *p* < 0.001 indicates high statistical significance).

The PROCESS macro model 6 of SPSS software was used to detect the chain-mediating effect of BF and DSES, as shown in Figure 1. After controlling for the interference of control variables in all regression equations, a significant positive correlation was observed between participants’ spirituality on BF levels (β = 1.4517, *p* < 0.001), DSES (β = 0.7115, *p* < 0.001), and SDM (β = 0.2733, *p* < 0.05). What is more, higher levels of BF and DSES were associated with higher SDM (β = 0.1391, *p* < 0.001 and β = 0.3704, *p* < 0.001). Additionally, a significant positive correlation between BF and DSES was found in patients (β = 0.2698, *p* < 0.001).

The bootstrap analysis revealed a direct effect of 0.2733 (BootSE = 0.1079, bootstrap 95% CI: 0.0611 to 0.4854) and a total indirect effect of 0.6106 (BootSE = 0.0924, bootstrap 95% CI: 0.4415 to 0.7959), with effect ratios of 30.9% and 69.1%, respectively. Importantly, there were three indirect pathways between spirituality and SDM: path 1, spirituality → BF → SDM (effect = 0.2020, BootSE = 0.0052, bootstrap 95% CI: 0.0058 to 0.0261); path 2, spirituality → DSES → SDM (effect = 0.2636, BootSE = 0.0042, bootstrap 95% CI: 0.0120 to 0.0284); and path 3, spirituality → BF → DSES → SDM (effect = 0.1451, BootSE = 0.0026, bootstrap 95% CI: 0.0061 to 0.0162), with an effect ratio of 22.9%, 29.8%, and 16.4%, respectively. The bootstrap 95% CI for the above effects did not include 0, indicating that they were statistically significant. The detailed results are shown in Table 5.

## 4. Discussion

In this study, we investigated patients with CHF who were receiving heart failure medications prior to reaching the standard dosage of these drugs. Four-step hierarchical regression analysis and bootstrapping were employed to test the hypothesized chain mediation model. The results of the data analysis illustrate the positive psychology categories of BF and decision self-efficacy as chain mediators between spirituality and SDM in patients with HF. A higher level of spirituality led to a higher level of SDM in patients with CHF (total effect = 0.8839, 95% CI = [0.7018, 1.0660]). This study reveals that spirituality has significant direct and indirect effects on SDM, and it also reveals the underlying psychological mechanisms from a positive psychology perspective for the first time.

### 4.1. The Positive Impact of Spirituality on SDM

The SDM score in patients with CHF was 62.12 ± 13.39, indicating a moderate level of participation. This result is consistent with Kou’s findings in coronary heart disease patients [48]. Interestingly, higher SDM participation in CHF patients with a class IV cardiac function classification was found for the first time. This phenomenon may be due to the complexity of drug treatment related to disease severity. CHF patients with class IV cardiac function classification are characterized by dyspnea at rest, and their daily life is seriously disturbed. Consequently, patients aspire to alleviate their symptoms and enhance their quality of life through medical treatment. This internal motivation encourages patients to obtain more anti-heart failure drug information from HCPs, thereby enhancing the degree of participation of both HCPs and patients. The results also support the core role of SDM in managing advanced HF in the heart failure guidelines [49]. In addition, the spiritual score of patients with CHF in this study was 26.43 ± 6.74, indicating a moderate level, which is consistent with previous findings [50]. Importantly, even after controlling for variables, spirituality was positively associated with SDM in patients with CHF (effect = 0.2733, 95% CI = [0.0611, 0.4854]). A qualitative study by Superdock also identified spirituality as a facilitator for individual SDM engagement [51]. This suggests that the sense of meaning in life embodied in spirituality may directly shape an individual’s core values, making them more likely to clarify their value preferences. Value clarification is a key element of SDM [45]. In addition, spiritual support has become a core area of palliative care for patients with CHF. Tobin RS noted that spirituality can reduce the mortality of HF. Currently, research on spirituality and SDM in patients with chronic diseases has been limited in confirming whether spiritual care through SDM is needed. This study investigates the relationship between spirituality and SDM, focusing on the potential impact of spirituality on SDM processes. Therefore, structured spiritual care services need to be considered in SDM practices. Before implementing SDM, HCPs need to undergo training in spiritual care techniques, such as spiritual history-taking tools, mindfulness-based approaches, or chaplaincy collaboration. At the same time, the inherent cultural burden of spirituality and SDM may influence individual beliefs, values, and communication patterns. In the future, we will explore the differences in the chain mediation effect in different cultural contexts, which will provide evidence for the development of decision aids with cultural characteristics.

### 4.2. The Independent Mediating Role of BF

The findings revealed that BF plays an independent mediating role between the level of spirituality and SDM in patients with HF (effect = 0.2020, 95% CI = [0.0058, 0.0261]). From the visual angle of positive psychology, BF is the process of exploring one’s potential in adversity and perceiving the beneficial aspects of stressful events through positive thinking [52]. Studies in oncology [53] and dying patients [54] have demonstrated a positive correlation between patient spirituality and BF, and that spirituality can lead to personal growth by guiding patients to find meaning and value in their lives. In this study, it was also revealed that spirituality exerts a positive influence on BF. BF plays a crucial role in the process of patients from disease cognition remodeling to health behavior decision-making. In this process, individuals seek positive meaning from the experience of illness and promote changes in health behaviors. Costa et al. [55] also proposed that BF is associated with positive health behavior change. When individuals face health challenges, positive changes in cognition can enhance patients’ sense of active participation in medical decision-making, and this positive response in cognitive behavior serves as the external manifestation of BF. However, no previous studies have directly demonstrated the impact of BF on the willingness of patients to participate in SDM. This study found that BF has a positive effect on SDM, which provides a new explanatory dimension for realizing real SDM. Obviously, it can be seen that patients with higher levels of spirituality may be more acutely aware of growth opportunities and potential value in their illness experience. This enhances their participation and enthusiasm in SDM. This study not only clarifies the effect of spirituality on SDM from the perspective of cognitive and behavioral mechanisms but also enlightens medical staff. Narrative intervention centered on humanistic care can be considered to elevate the level of spirituality, and then the potential for patient growth in experiencing illness can be explored, ultimately realizing true SDM. The study also showed that narrative intervention promotes individuals to find resilience resources and thus actively cope with the disease [56].

### 4.3. The Mediating Role of Decision Self-Efficacy

Decision self-efficacy mediated the association between spirituality and SDM (effect = 0.2636, 95% CI = [0.0120, 0.0284]). At present, the relationship between spirituality and decision self-efficacy has primarily been explored in caregivers of individuals with chronic diseases. Dionne-Odom et al. [57] found that spiritual growth among family caregivers of cancer patients with poor prognosis exhibited a positive correlation with decision self-efficacy. The scholar pointed out that individuals with lower levels of spirituality may struggle to explore meaning, value, and purpose in life, which can limit their confidence or belief in their ability to actively participate in SDM. In 2017, a revised three-talk model of SDM divided decision-making into three stages: antecedent, process, and result. Decision self-efficacy is a key factor in the antecedent stage [58]. In clinical studies of SDM, decision self-efficacy has emerged as a primary outcome of intervention. Previous studies indicated that decision-making efficiency affected participation in SDM [45]. The role of family decision self-efficacy in SDM has gradually garnered attention. In a structural equation modeling study of eHealth literacy and SDM, Nejati et al. [59] found that family decision self-efficacy was a factor that independently influenced SDM. This was the first study to identify the mediating role of decision self-efficacy between spirituality and SDM in patients with chronic disease. The study provided evidence for the incorporation of spirituality interventions into SDM. Enhanced spirituality facilitates the activation of subjective decision-making awareness and strengthens decision self-efficacy, thereby ultimately promoting SDM. In the future, it is necessary to carry out relevant empirical research on interventions.

### 4.4. The Chain-Mediating Role of BF and Decision Self-Efficacy

The research confirmed that BF and decision self-efficacy play a chain-mediating role in the relationship between spirituality and SDM (effect = 0.1451, 95% CI = [0.0061, 0.0162]). This study found that patients with higher levels of spirituality were more likely to exhibit positive cognitive and behavioral coping mechanisms in adverse situations. Enhanced BF can facilitate greater participation in SDM by improving decision self-efficacy. Self-efficacy plays an important role in attitudes, emotions, and health management behaviors [60]. Rezori et al. [61] found that BF was positively correlated with self-efficacy, and BF could predict the level of self-efficacy. Throughout the disease process, patients can experience personal growth through sense-making, which enhances their confidence in decision-making abilities and encourages active participation in SDM. This study reveals the chain mechanism through quantitative research for the first time, which enriched people’s understanding of the SDM mechanism.

### 4.5. Practical Implications

This study provided valuable data for the strategy optimization of drug management in HF and updating guidelines of HF management in the future. In developing patient decision aid (PDA) for anti-heart failure medication, this study suggests that assessment and intervention of spirituality and BF need to be included. This innovation not only facilitates SDM but also enhances the effect of SDM in medication adherence. In addition, this study fills a gap on the psychological cognitive mechanism of how spirituality affects SDM. It provides a theoretical basis for the intervention of hope and meaning in life, as well as the application of cultural competence care technology with narrative characteristics in SDM. The field of medical and health education should pay attention to the cultivation of students’ spiritual intelligence. With the coming of “transcendent AI,” some scholars have proposed that it is necessary to establish a dynamic mapping mechanism between clinical decision-making and individual value systems by employing knowledge graph technology [62]. Balancing the relationship between the meaning of human life and technology will also become the focus of future healthcare systems.

### 4.6. Limitations

However, this study has some limitations. Firstly, this study has a cross-sectional quantitative design, and static data cannot reflect the time effect. In the future, mixed studies, longitudinal studies, and intervention studies are recommended to further explore the interactions among variables, such as observing the long-term effects of strategies to improve BF and decision self-efficacy on SDM in patients with HF. Secondly, since the sample source of this study was a single provincial coastal medical institution, there might be a potential selection bias. Therefore, it is suggested that multi-center clinical trials be carried out in the future. Thirdly, this study employed convenience sampling, which may have potential limitations of sample representativeness.

## 5. Conclusions

Spirituality not only directly influences shared decision-making but also indirectly facilitates SDM through enhancing BF, as well as the chain-mediated effects of BF and decision self-efficacy. Therefore, in the SDM program of patients with HF, we need to consider stimulating spirituality, BF, and decision self-efficacy, which can promote the level of shared decision-making. We suggest that it is necessary to conduct an intervention of these variables in the process of SDM, which can further validate the mechanistic model. This may provide innovative strategies for improving medication adherence and enhancing long-term health outcomes in heart failure patients.

## Figures and Tables

**Table 1:** Characteristics of participants and difference in spirituality, BF, DSES, and SDM (*n* = 387).

Characteristics	Categories	*n* (%)	Spirituality			BF			DSES			SDM		
			M ± SD	*t*/*F*	*p*	M ± SD	*t*/*F*	*p*	M ± SD	*t*/*F*	*p*	M ± SD	*t*/*F*	*p*
Gender	Male	256 (66.1)	26.26 ± 6.90	0.682	0.496	59.05 ± 16.94	−0.778	0.437	62.80 ± 14.79	−0.543 ^a^	0.588	62.84 ± 14.25	−1.586 ^a^	0.114
	Female	131 (33.9)	26.76 ± 6.42			57.68 ± 15.02			62.00 ± 13.18			60.72 ± 11.45		
Age (years)	≤59	103 (26.6)	25.62 ± 6.30	0.770	0.511	56.74 ± 17.29	1.335	0.263	61.53 ± 15.22	4.413	0.005	61.80 ± 16.39	0.779 ^a^	0.506
	60–69	125 (32.3)	26.97 ± 7.19			59.93 ± 15.57			65.49 ± 13.81			63.01 ± 12.86		
	70–79	112 (28.9)	26.52 ± 6.59			59.85 ± 15.11			62.45 ± 13.31			62.48 ± 11.39		
	≥80	47 (12.1)	26.55 ± 6.84			56.03 ± 18.51			57.03 ± 13.90			59.61 ± 11.91		
Marital status	Single	73 (18.9)	26.05 ± 6.78	0.526	0.599	59.00 ± 17.11	−0.243	0.808	61.07 ± 13.45	0.972	0.332	60.43 ± 14.18	1.200	0.231
	Others	314 (81.1)	26.52 ± 6.74			58.49 ± 16.15			62.87 ± 14.43			62.52 ± 13.20		
Address	Urban	196 (50.6)	25.56 ± 6.26	2.585	0.010	57.61 ± 16.17	1.198	0.232	61.05 ± 13.58	2.073	0.039	61.95 ± 13.80	0.257	0.797
	Rural	191 (49.4	27.32 ± 7.11			59.59 ± 16.44			64.05 ± 14.80			62.30 ± 13.00		
Education	Junior school and below	125 (32.3)	25.35 ± 7.42	2.384	0.094	61.29 ± 17.66	3.555 ^a^	0.030	61.15 ± 14.46	0.864 ^a^	0.422	60.55 ± 15.12	1.631 ^a^	0.197
	Senior school	218 (56.3)	26.92 ± 6.34			56.67 ± 14.86			63.18 ± 13.41			62.55 ± 11.61		
	College and above	44 (11.4)	27.07 ± 6.42			60.42 ± 18.24			63.24 ± 17.46			64.44 ± 16.05		
Monthly income (CNY)	≤5000	185 (47.8)	25.66 ± 6.21	2.160	0.031	57.74 ± 16.18	0.976	0.330	61.17 ± 13.58	1.806	0.072	62.10 ± 14.01	0.025	0.980
	>5000	202 (52.2)	27.13 ± 7.13			59.36 ± 16.43			63.78 ± 14.77			62.14 ± 12.84		
Religious	YES	41 (10.6)	25.66 ± 7.50	−0.774	0.440	59.89 ± 19.60	0.541	0.589	62.11 ± 16.02	−0.201	0.841	63.59 ± 14.33	0.740	0.460
	NO	346 (89.4)	26.52 ± 6.65			58.43 ± 15.90			62.58 ± 14.06			61.95 ± 13.29		
BMI	<18.5	21 (5.4)	26.05 ± 7.47	0.859	0.489	52.85 ± 18.80	1.192	0.314	60.10 ± 15.18	1.390	0.237	60.32 ± 15.37	1.076	0.368
	18.5–22.9	123 (31.8)	26.76 ± 6.51			59.78 ± 17.07			61.77 ± 14.88			62.91 ± 13.92		
	23–24.9	95 (24.5)	25.78 ± 6.24			58.44 ± 15.01			63.91 ± 13.05			63.16 ± 11.92		
	25–29.9	131 (33.9)	26.91 ± 7.29			59.09 ± 16.14			63.43 ± 14.11			61.62 ± 13.02		
	≥30	17 (4.4)	24.41 ± 5.76			53.96 ± 15.39			56.39 ± 15.42			56.75 ± 17.16		
Duration of disease	≤12	198 (51.2)	27.17 ± 7.07	1.767	0.153	58.73 ± 16.40	1.382	0.248	62.89 ± 14.54	0.104	0.958	62.12 ± 13.44	3.029	0.029
	13–24	62 (16.0)	25.89 ± 6.29			60.67 ± 14.65			61.91 ± 13.77			64.97 ± 13.73		
	25–36	54 (14.0)	25.87 ± 6.09			59.94 ± 15.40			62.50 ± 13.75			63.75 ± 12.27		
	≥37	73 (18.9)	25.29 ± 6.54			55.43 ± 17.85			62.10 ± 14.49			58.50 ± 13.22		
Smoker	YES	206 (53.2)	26.75 ± 6.80	0.992	0.322	59.10 ± 16.12	0.662	0.509	62.58 ± 14.05	0.074	0.941	61.48 ± 13.12	−1.001	0.317
	NO	181 (46.8)	26.07 ± 0.67			58.00 ± 16.56			62.47 ± 14.52			62.85 ± 13.70		
HUA	YES	27 (7.0)	24.19 ± 7.30	1.799	0.073	57.14 ± 18.18	0.477	0.633	57.07 ± 15.36	2.071	0.039	60.33 ± 13.52	0.722	0.471
	NO	360 (93.0)	26.60 ± 6.68			58.69 ± 16.19			62.94 ± 14.11			62.26 ± 13.39		
HR	<100	325 (84.0)	26.19 ± 6.75	−1.625	0.108	58.06 ± 16.15	−1.457	0.146	62.00 ± 14.03	−1.689	0.092	61.91 ± 13.12	−0.700	0.484
	≥100	62 (16.0)	27.68 ± 6.57			61.35 ± 17.00			65.33 ± 15.20			63.21 ± 14.80		
HBP	YES	197 (50.9)	26.48 ± 6.23	−0.143	0.886	58.55 ± 15.99	0.041	0.967	62.98 ± 14.22	−0.633	0.527	62.16 ± 12.63	−0.063 ^a^	0.950
	NO	190 (49.1)	26.38 ± 7.25			58.62 ± 16.68			62.06 ± 14.31			62.08 ± 14.17		
HLD	YES	132 (34.1)	25.96 ± 6.53	0.980	0.328	56.41 ± 15.47	1.894	0.059	62.92 ± 13.69	−0.390	0.697	62.40 ± 12.54	0.297	0.767
	NO	255 (65.9)	26.67 ± 6.85			59.71 ± 16.65			62.33 ± 14.56			61.98 ± 13.84		
HG	YES	94 (24.3)	26.59 ± 6.81	−0.258	0.797	60.14 ± 15.46	−1.061	0.289	62.05 ± 15.14	0.378	0.706	63.07 ± 13.61	−0.791	0.430
	NO	293 (75.7)	26.38 ± 6.73			58.09 ± 16.57			62.69 ± 13.98			61.82 ± 13.33		
NYHA	2	99 (25.6)	26.79 ± 6.63	1.634	0.197	55.79 ± 16.32	1.976	0.140	61.69 ± 15.21	0.931	0.395	59.22 ± 13.89	4.011	0.019
	3	190 (49.1)	26.79 ± 6.63			59.64 ± 16.63			63.53 ± 13.99			62.39 ± 13.01		
	4	98 (25.3)	25.37 ± 7.00			59.37 ± 15.50			61.44 ± 13.77			64.53 ± 13.22		

Note: BF, benefit-finding; DSES, decision self-efficacy; SDM, shared decision-making; BMI, body mass index; HUA, hyperuricemia; HR, heart rate; HBP, high blood pressure; HLD, hyperlipidemia; HG, hyperglycemia; NYHA, New York Heart Association. *t*, *t*-test; *F*, one-way ANOVA analysis. ^a^, Welch’s *t*-test or Welch’s ANOVA.

**Table 2:** Descriptive statistics of target variables.

Variables	Range	M ± SD	Standardized Score (M ± SD)
Spirituality	11–46	26.43 ± 6.74	2.20 ± 0.56
Benefit-finding	22–103	58.59 ± 16.31	2.66 ± 0.74
Decision self-efficacy	27.88–96.88	62.53 ± 14.26	5.68 ± 1.30
Shared decision-making	27.43–92.63	62.12 ± 13.39	6.90 ± 1.49

Note: M = mean; SD = standard deviation.

**Table 3:** Partial correlations between target variables after controlling for potential confounders.

Variables	Mean ± SD	1	2	3	4
1. Spirituality	26.43 ± 6.74	1			
2. Benefit-finding	58.59 ± 16.31	0.570 **	1		
3. Decision self-efficacy	62.53 ± 14.26	0.523 **	0.500 **	1	
4. Shared decision-making	62.1 2 ± 13.39	0.428 **	0.450 **	0.546 **	1

** *p* < 0.01 (two-tailed).

**Table 4:** Hierarchical regression analysis of spirituality, benefit-finding, and decision self-efficacy on predicting shared decision-making.

Variables	Block 1		Block 2		Block 3		Block 4	
	B (SE)	β	B (SE)	β	B (SE)	β	B (SE)	β
Address	−2.791 (4.121)	−0.104	0.067 (3.716)	0.002	0.119 (3.587)	0.004	0.429 (3.332)	0.016
Income	2.409 (4.175)	0.090	1.275 (3.754)	0.048	0.871 (3.624)	0.033	1.348 (3.367)	0.050
Education	2.439 (1.112)	0.114 *	1.536 (1.004)	0.072	2.231 (0.977)	0.105 *	1.857 (0.909)	0.087 *
Age	−0.618 (0.714)	−0.045	−0.829 (0.642)	−0.061	−0.723 (0.620)	−0.053	−0.088 (0.582)	−0.006
HUA	−1.409 (2.670)	−0.027	0.900 (2.412)	0.017	0.390 (2.330)	0.007	1.962 (2.173)	0.037
Duration of disease	−0.715 (0.569)	−0.063	−0.140 (0.515)	−0.012	−0.174 (0.497)	−0.015	−0.345 (0.462)	−0.031
NYHA	3.156 (0.977)	0.168 ***	3.882 (0.882)	0.207 ***	3.248 (0.859)	0.173 ***	3.202 (0.798)	0.171 ***
Spirituality			0.884 (0.093)	0.445 ***	0.537 (0.110)	0.270 ***	0.273 (0.108)	0.137 *
Benefit-finding					0.239 (0.045)	0.291 ***	0.139 (0.043)	0.169 ***
Decision self-efficacy							0.370 (0.047)	0.394 ***
*F*	2.409 *		13.994 ***		16.554 ***		23.367 ***	
Adjusted R^2^	0.025		0.212		0.266		0.367	
ΔR^2^	0.043		0.186		0.055		0.100	

Abbreviations: HUA, hyperuricemia; NYHA, New York Heart Association. * *p* < 0.05; *** *p* < 0.001.

**Table 5:** Bootstrap direct and indirect mediating effects.

			95% CI		
Paths	Effect	Boot SE	Boot LLCI	Boot ULCI	Effect Ratio (%)
Total effect	0.8839	0.0926	0.7018	1.0660	
Direct effect	0.2733	0.1079	0.0611	0.4854	30.9
Indirect effect	0.6106	0.0924	0.4415	0.7959	69.1
Path 1: Spirituality → BF → SDM	0.2020	0.0052	0.0058	0.0261	22.9
Path 2: Spirituality → DSES → SDM	0.2636	0.0042	0.0120	0.0284	29.8
Path 3: Spirituality → BF → DSES → SDM	0.1451	0.0026	0.0061	0.0162	16.4

Abbreviations: BF, benefit-finding; DSES, decision self-efficacy; SDM, shared decision-making.

## Data Availability

The data analyzed in this study are available on reasonable request to the corresponding author.

## Abbreviations

The following abbreviations are used in this manuscript:

HF	Heart failure
BF	Benefit-finding
SDM	Shared decision-making
